# How do macro-level structural determinants affect inequalities in mental health? – a systematic review of the literature

**DOI:** 10.1186/s12939-018-0879-9

**Published:** 2018-12-06

**Authors:** A. McAllister, S. Fritzell, M. Almroth, L. Harber-Aschan, S. Larsson, B. Burström

**Affiliations:** 1 Department of Public Health Sciences, Karolinska Institutet, Stockholm, Sweden; 2 The Public Health Agency of Sweden, Stockholm, Sweden

**Keywords:** Mental health, Structural determinants, Systematic review, Inequalities, Gender, Socio-economic, Equity

## Abstract

**Background:**

In Europe and elsewhere there is rising concern about inequality in health and increased prevalence of mental ill-health. Structural determinants such as welfare state arrangements may impact on levels of mental health and social inequalities. This systematic review aims to assess the current evidence on whether structural determinants are associated with inequalities in mental health outcomes.

**Methods:**

We conducted a systematic review of quantitative studies published between 1996 and 2017 based on search results from the following databases Medline, Embase, PsychInfo, Web of Science, Sociological Abstracts and Eric. Studies were included if they focused on inequalities (measured by socio-economic position and gender), structural determinants (i.e. public policies affecting the whole population) and showed a change or comparison in mental health status in one (or more) of the Organisation for Economic Cooperation and Development (OECD) countries. All studies were assessed for inclusion and study quality by two independent reviewers. Data were extracted and synthesised using narrative analysis.

**Results:**

Twenty-one articles (17 studies) met the inclusion criteria. Studies were heterogeneous with regards to methodology, mental health outcomes and policy settings. More comprehensive and gender inclusive welfare states (e.g. Nordic welfare states) had better mental health outcomes, especially for women, and less gender-related inequality. Nordic welfare regimes may also decrease inequalities between lone and couple mothers. A strong welfare state does not buffer against socio-economic inequalities in mental health outcomes. Austerity measures tended to worsen mental health and increase inequalities. Area-based initiatives and educational policy are understudied.

**Conclusion:**

Although the literature on structural determinants and inequalities in mental health is limited, our review shows some evidence supporting the causal effects of structural determinants on mental health inequalities. The lack of evidence should not be interpreted as lack of effect. Future studies should apply innovative methods to overcome the inherent methodological challenges in this area, as structural determinants potentially affect both levels of mental health and social inequalities.

**Electronic supplementary material:**

The online version of this article (10.1186/s12939-018-0879-9) contains supplementary material, which is available to authorized users.

## Introduction

The burden and prevalence of mental ill-health and mental illnesses are increasing [1]. Research shows that there are many explanations for this such as better awareness and diagnosis, environmental factors, structural factors and changes in public policy [2]. In this review, we focus on the structural, defining structural determinants as public policies affecting the whole population [3], we propose six main domains of welfare states, family policy, employment policy, income support and social insurance policy, area-based initiatives and education policy (see further explanation below). The Swedish Government commissioned The Public Health Agency of Sweden to increase the knowledge on mental health inequalities and their underlying determinants, this study is part of this larger project. Against this background, the main focus of the review was on studies of structural determinants and policies in Western welfare states. We define mental health broadly including positive mental health, mental ill-health and diagnoses of mental illnesses. Overall, we aim to investigate whether structural determinants are associated with mental health outcomes and if these determinants differentially impact on mental health outcomes by socio-economic status (SES) and gender.

The following provides a further explanation of inequalities in mental health and structural determinants of mental health.

### What are inequalities in mental health?

The burden of mental illness is not equally distributed in the population. Epidemiological evidence consistently demonstrates an inverse association between SES and psychiatric morbidity, such that more disadvantaged groups are affected by mental illness to a greater extent [4]. Also, demographic factors such as gender and ethnicity (although not in themselves modifiable) may further modify the risk of mental disorder, depending in turn on how wealth, power and resources are distributed by gender and ethnicity (see for example [5, 6]). This further suggests that distributions of mental illness are systematically shaped by social, economic as well as physical environments throughout the life-course [7], putting more disadvantaged population sub-groups at greater risk for mental illnesses through exposure to unfavourable social and economic circumstances.

### How are structural determinants related to mental health?

We propose that structural determinants affect the distribution of resources and have the potential to influence mental health inequalities. Previous research shows that welfare state arrangements, social and economic policy may influence the distribution of health between social groups [3, 8–12]. We used this literature to deconstruct structural determinants into six public policy domains: welfare states, family policy, employment policy, income support and social insurance policy, area-based initiatives and education policy (see Table 1). Borrell*,* et al. [3] suggest that such policy domains are drivers of the social structure and power relations that ultimately generate social inequalities in health. We suggest that these policy domains may mitigate or reduce the risk of poor mental health that provides the conditions for everyday life and influence the opportunities available to people across the life course. We also acknowledge the importance of healthcare policies in shaping access to services, and that these are likely to contribute to mental health inequalities. However, we do not asses these in this review as we conceptualise these as downstream factors influencing the treatment of mental illnesses, as opposed to broader structural determinants of mental illness.

**Table 1:** Policy domains and examples

Policy domain	Explanation and examples
1. Welfare state	Typologies of welfare states, based on family policy, social policy or other dimensions.
2. Family policy	Levels of benefits, changes in eligibility, coverage, public daycare, custody laws, parental leave.
3. Employment policy	Minimum wage, flexibility, precariousness, tax-credits/subsidies, active labour market policies, employment protection legislation, anti-discrimination law, strength of unions.
4. Income support and social insurance	Levels of benefits (including unemployment), changes in eligibility, coverage.
5. Area-based initiatives	Affordable housing, availability, subsidies, regulations on eviction, quality of housing, neighbourhood renewal.
6. Education (at all levels)	Affordability, access, developmental support.

We included all welfare typologies in our review. We propose, that regardless of the typology, examining welfare regimes provides insight into the values and norms that influence structural determinants. To illustrate, we use Korpi’s [13] family model regime typology. The dual-earner/carer models (e.g. Denmark, Sweden, Norway, Finland) are characterised by providing universalistic public policies to encourage labour force participation and gender equality. In contrast, the market-oriented model (e.g. Australia, the United Kingdom and the United States) provides limited social protection mostly towards those considered ‘deserving’ through means-tested benefits. In this welfare regime, the market, rather than public policy determines gender roles. In traditional family models, (e.g. Belgium, the Netherlands, Spain), policies are organised around the family with men often viewed as the ‘breadwinner’. Unpaid labour is seen as a responsibility of the family rather than the state which leads to low support for female labour force participation.

While social determinants of mental illness have long been recognised [7, 14], only recently have they received more attention, especially in the wake of the recent economic crisis [15]. However, most empirical research focuses on proximal, “down-stream” determinants and few focus on broader, “upstream”, what we define as structural determinants and how these might affect social distributions of mental illness. To the best of our knowledge, no systematic review of the literature exists on structural determinants and their impact on mental health outcomes. We specifically sought to answer the following:

1. Which structural determinants (i.e. public policies) are associated with inequalities in mental health outcomes?
2. In what context have these policies been implemented?
3. What social differentials (across socio-economic groups and between men and women) exist regarding mental health outcomes?

## Methods

This review was structured in accordance with the Preferred Reporting Items for Systematic Reviews and Meta-Analyses (PRISMA) guidelines [16], with additional focus on equity using the PRISMA-E 2012 [17].

### Information sources and search strategy

We searched for eligible articles in the following databases: Medline (Ovid), Embase (Embase.com), PsycINFO (Ovid), Web of Science, Sociological Abstracts (ProQuest) and ERIC (ProQuest). The Karolinska Institutet Library completed the initial search on 16 March 2017 and an updated search on 7 November 2017.

We also reviewed publications of recognised experts in this area as well as other relevant studies and projects such as Evaluating the Impact of Structural Policies on Health Inequalities (SOPHIE) [18]. Two reviewers also screened the bibliographies of all relevant reviews. See Additional file 1 for a detailed search strategy. Ethical approval was not required as results are based on previously published papers.

### Eligibility criteria

Articles were considered eligible if they were (1) Original, peer-reviewed, written in English and published between 1996 and 2017. (2) Quantitative studies showing a change or comparison in mental health status (i.e. mental disorder diagnosis, positive (self-rated) mental health, suicide rate) using a validated mental health measure, and (3) Examined one of the policy domains (Table 1) in at least one of the Organisation for Economic Cooperation and Development (OECD) countries. See Additional file 2 for more information about the inclusion and exclusion criteria used in the selection process.

### Study selection

We employed two levels of screening to identify relevant studies. All screening tools were pilot tested before each level of screening. In the following section, we briefly describe each level:

#### Level 1 – Title and abstract screening

Most articles were excluded at this level because the title and abstract did not focus on mental health and/or one of the policy domains. This was primarily conducted by SL. Another reviewer (AM) independently applied the criteria for inclusion and exclusion to 14% of the title and abstracts (350 references). Any disagreements between SL and AM were resolved after discussions.

#### Level 2 – Full-text screening

The selection criteria were clarified and rewritten for the Level 2 – Full-text screening. Four reviewers participated in Level 2 screening. All articles were reviewed by at least two reviewers. The review team discussed articles where there were disagreements on final decisions (30%). If the team could not agree, then the article was reviewed by a third member of the review team. AM was responsible for making all final decisions.

### Study quality assessment and risk of bias

All included articles were assessed using the “Health Evidence Bulletin, Wales: Questions to assist with the critical appraisal of an observational study” (hereto referred to as HEB – Wales Tool) [19]. The HEB – Wales Tool is one of the few quality assessment tools that is designed to fairly assess different study designs. The tool has been endorsed by Sanderson*,* et al. [20] for its ability to be used to assess cohort, case-control and cross-sectional study designs; transparency regarding development; applicability for future use; and use of a checklist system which we used to develop a rating scale. We adapted the tool so that questions were most relevant to our study aims (see Additional file 3).

While the HEB – Wales Tool was designed as a checklist rather than a scoring tool, we agreed on a scoring system (a priori) where the study was given a score of two if the criteria for the item was met. If it was unclear then a score of one was given, and if it clearly did not meet the criteria then a score of zero was given. Each study was given a total score out of 30 possible points. If a study had a score of more than 23 points (the authors met at least 80% of the items), then it was classified as high quality. Medium quality studies had a cut-off score between 18 to 23 points and low-quality studies scored 17 points or less (the authors only met 60% of the items). All articles were quality appraised by two reviewers. The final score is an average of the two reviewers’ scores (see Additional file 4 for a summary of the scores).

We assessed the risk of bias in individual studies using Part B of the HEB – Wales Tool entitled “Do I trust it?” In this section, we assessed whether the study design and study population was appropriate, confounding and bias were considered in the study, and there was a long enough follow-up time. Most of the risk of bias was assessed at the design rather than outcome level.

### Data extraction strategy

A data extraction template was created and piloted by SF and AM. Using this template, we extracted data from all articles that were marked as ‘included’ in Level 2 screening. Four reviewers completed this phase, with two reviewers assigned to each article to extract data. AM then compared the results and completed a summary table. Any discrepancies were resolved through discussion between the two reviewers.

### Data items

Table 2 summarises the data items extracted from each article.

**Table 2:** Data items from each article

Article information	Author, year, aim, setting (i.e. country)
Characteristics of study	Study population, sample size, method of data collection and year(s) covered
Exposures	Policy domains (1–6), policy sub-area, name of policy
Outcome measures	Mental health aspect, mental health (validated) measurement used, type of inequality
Inequality measure	Assessed socio-economic status or gender inequality (increase/decrease/neutral)

### Data synthesis

We used narrative analysis to synthesise the data. Categorising the policy domains and focusing on the two specific types of inequalities (gender and SES), were deliberate strategies intended to make the data synthesis clearer. Furthermore, other data items extracted such as study design, population, and setting were intentional measures to assist with comparing heterogeneity in the studies. The implementation of the HEB-Wales Tool also made it possible to systematically compare the quality of the studies.

## Results

### Study selection

**Fig. 1:**
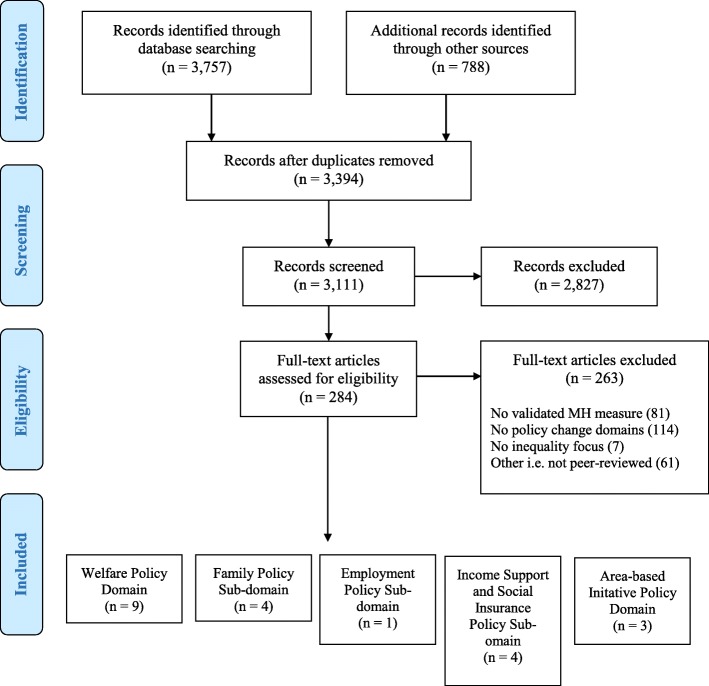
PRISMA Flow Diagram [15]

The search strategy and other sources produced 3394 papers which were assessed for inclusion in the review. Data were extracted from 21 papers that met our eligibility criteria. Figure 1 shows a PRISMA flow diagram of the selection process.

### Study characteristics

The 21 selected articles were representative of 17 different studies or data sources. The majority investigated European countries, including 10 articles involving Sweden. Five articles, however, included data from Australia, Canada, the USA and Japan. Twelve of the studies compared two or more countries, while the remaining nine focused on a single country. One study focused on adolescents and the remaining 20 involved a working age population^1^.

Thirteen articles used cross-sectional methods and 10 used longitudinal methods, two of which used a natural policy experiment design.

The articles measured constructs of positive mental health, mental ill-health and diagnoses of mental illnesses. Positive mental health constructs were represented in 11 articles and included mental health functioning, mental well-being and social-emotional development. Mental distress, poor mental health, depression, suicide rates, psychiatric diagnosis and anti-depressant prescription represent the negative aspects of mental health and diagnoses of mental illnesses measured in 10 of the studies. Several validated mental health measures were used including the World Health Organization Well-Being Index (WHO-5), the Global Health Questionnaire (GHQ-12), the Short Form Health Survey (SF36 and SF12), the Centre for Epidemiological Studies Depression Scale (CES-D8), Mental Health Inventory (MHI-5), Ages and Stages Questionnaire (ASQ-SE), Health Behaviour in School Age Children (HBSC), Self-Reported Health in the Quarterly Labour Force Survey, suicide statistics and register data for psychiatric diagnoses.

Five articles focused only on gender inequalities in mental health, and 12 articles measured only SES inequalities, with four investigating both types of inequalities. Table 3 summarizes the results from the 21 included articles.

**Table 3:** Summary of results

Article	Aim	Study population (sample size); Study design; Country	Policy sub-area	MH aspect *(measure)*	Type of inequality assessed Change in inequality
Policy domain – Welfare States					
Artazcoz L, Cortès I*,* et al. 2013. [21] +++	Analyse the relationship between health status, paid working hours and household composition across family policy typologies.	25–64 years (only those employed and partnered) (*N*=19,364) Cross-sectional 27 European countries^b^	Family policy	Mental well-being *(WHO-5)*	Focus: Gender inequalities (Korpi’s welfare regime typology) Dual-earner/dual-carer models had better mental health outcomes for both sexes than other family policy models.
De Moortel D, Palència L*,* et al. 2015. [22] +++	Investigate across welfare regimes, the association between neo-Marxian social class (NMSC) and employee mental well-being and identify any gender differences.	15–65 years (*N*=14,107) Cross-sectional 21 European countries^b^	Employment, social insurance and gender policies	Mental well-being *(WHO-5)*	Focus: Gender and SES inequalities (NMCS) (Korpi’s welfare regime typology) Across all welfare regimes, men reported better mental well-being than women and this difference is stronger in lower SES. Inequalities in mental well-being related to SES was found in Contradictory, State corporatist/family support and Southern welfare regimes but not in Basic security-market oriented welfare regime. No SES differences in mental well-being among women in dual earner models but differences in men.
De Moortel D, Vandenheede H*,* et al. 2014. [23] ++	Assess whether measures of employment quality are related to mental well-being and if this relationship differs by gender and across different welfare models.	15–65 years employed (*N*=12,271) Cross-sectional 21 European countries^b^	Employment conditions	Mental well-being *(WHO-5)*	Focus: Gender inequalities (Korpi’s welfare regime typology) Gender differences in mental well-being are least pronounced in earner-carer countries.
Niedzwiedz CL, Mitchell RJ*,* et al. 2016. [24] +++	Investigate whether spending on 3 types of social protection (unemployment, ALMPs, family) influences social inequality in depressive symptoms.	20–64 years (*N*=48,397) Cross-sectional 18 European countries^b^	Unemployment, ALMPs, family policy	Depression *(CES-D8)*	Focus: SES inequalities Decrease in benefit levels or increased conditionality may decrease mental health of disadvantaged groups. Increase in social protection may reduce inequalities in depressive symptoms.
Nordenmark M, Strandh M*,* et al. 2006. [25] +++	Investigate the impact of unemployment benefit system for mental well-being in different welfare regimes.	Unemployed persons (*N* = 3442) Longitudinal (cohort) Britain, Ireland and Sweden	Unemployment benefits	Mental distress *(GHQ-12)*	Focus: SES inequalities (Esping-Andersen’s weflare typology) Welfare regime has significant impact on mental well-being of the unemployed. Inverse class gradient in mental well-being in Ireland and UK compared to a positive class gradient in mental well-being in Sweden. Flat-rate benefits decrease the mental health of those with higher SES. Income replacement benefits tend to maintain pre-unemployment differences in mental distress.
Sekine M, Chandola T*,* et al. 2009. [26] +++	Investigate socio-economic differences in work characteristics and health in Finland, Japan and the UK.	40–60 years (civil servants) (*N*=17,801) Longitudinal (cohort) Finland, UK, Japan	Employment conditions and work characteristics	Mental health functioning *(SF-36)*	Focus: SES inequalities Japanese males, lower SES tended to have poorer mental health functioning. No consistent SES differences in mental health functioning were observed among British and Japanese women. Finnish men and women, higher SES had poor mental health functioning.
Sekine M, Tatsuse T*,* et al. 2011. [27] +++	Investigate whether work characteristics contribute to sex inequalities in health in liberal, social democratic and conservative welfare states.	40–60 years (civil servants) (N=17,801) Longitudinal (cohort) Finland, UK, Japan	Employment conditions and work characteristics	Mental health functioning *(SF-36)*	Focus: Gender inequalities Poor mental health functioning was largest among Japanese women, followed by British women, then Finnish women. Sex differences in mental health functioning were the smallest in the Finnish population.
Van de Velde S, Bambra C*,* et al. 2014. [28]^a^ +++	Examine whether there are smaller inequalities between lone and cohabitating mothers in welfare regimes with higher levels of universalism and policies targeted at defamilising.	Women 18–55 years w/ children aged 18 years or younger (*N*=26,499)^a^ Cross-sectional 27 European countries^b^	Unemployment, family policy	Depression *(CES-D 8)*	Focus: Gender inequalities (Ferrera’s welfare typology) and SES Larger mental health differences between lone and cohabitating mothers in Britain than in Sweden. Welfare regime seems to moderate inequalities in mental health between lone and cohabitating mothers. Lowest inequalities found in Nordic welfare regimes and largest in Bismarckian regimes. Relationship between measures of SES and mental health among lone mothers according to welfare state less clear, but Bismarckian and Nordic models were more equal according to education level.
Yur’yev A, Värnik A*,* et al. 2012. [29] ++	Assess the relationship between suicide mortality and social expenditure.	European countries; (*N*= 26) Time trends, Ecological 26 European countries^b^	Social expenditure	Suicide *(S)*	Focus: No explicit inequality focus but analysis is stratified by sex Increased social expenditure associated with decrease in female suicides in most countries.
*Sub-domain: Family policy*					
Chandola T, Martikainen P*,* et al. 2004. [30] +++	Examine whether welfare states with more family friendly workplace policies mitigates the effect of work and family conflict on mental health and whether there are differences between men and women.	Employed aged 35–60 years (*N*=14,706) Cross-sectional Finland, Japan, UK	Family-friendly workplace policies	Mental health functioning *(SF-36 MCS)*	Focus: Gender inequalities Single fathers in all 3 countries, single mothers in Finland had poorer mental health compared to other family arrangements. But Finnish men and women had better mental health and less conflict between work and family than the other countries. Welfare states with more family-friendly workplace policies may reduce inequalities in mental health for women, except for single parents.
Hewitt B, Strazdins L*,* et al. 2017. [31] +++	Investigate the health effects of the introduction of a new universal paid parental leave (PPL) scheme in Australia.	Employed mothers (*N*=5615) (2347 pre-PPL, 3268 post-PPL) Cross-sectional, longitudinal Australia	Paid parental leave	Mental well-being *(SF-12)*	Focus: SES inequalities The Scheme improved mental health of all mothers but did not reduce gap in SES inequalities among mothers.
Huang J, Kim Y*,* et al. 2017. [32] ++	Examine whether an economic intervention that encourages families to accumulate assets, reduces the social-emotional inequalities between children of unmarried mothers versus married mothers.	Mothers 18 years plus (*N*=2121) Natural policy experiment - Longitudinal USA	Child Development Accounts (CDA)	Emotional development *(ASQ-SE)*	Focus: SES (single mothers as a proxy for low SES) CDA have positive effects on social-emotional development for children living with unmarried mothers. Intervention could reduce mental health inequalities between children of unmarried and married mothers.
Rathmann K, Pförtner T-K*,* et al. 2016. [33] +++	Examine whether increased public spending relates to lower prevalence of mental health complaints and buffers against inequalities among adolescents.	Adolescents aged 11, 13 and 15-year-olds (*N*=144,754) Cross-sectional 27 European countries^b^	Family benefits	Psychological health complaints *(HSBC symptom checklist)*	Focus: SES (Family Affluent Scale) Social protection, especially family benefits, is positively linked to better overall mental health among young people. Increase in family benefits widened social inequalities during economic recession.
*Sub-domain: Employment*					
Andersen I, Brønnum-Hansen H*,* et al. 2016 [34] +++^c^	Study the impact of ALMP and stricter eligibility criteria for income support among people with chronic illness, on their employment rate and receipt of non-health related benefits.	Residents aged 20–60 years (N=2,778,044) Register-based cohort, cross-sectional Denmark	ALMPs and income support	Psychiatric diagnosis *(Hospital records and anti-depressant, anxiolytic & neuroleptic prescriptions)*	Focus: Gender and SES inequalities Increase in mental health problems for those in lower SES group after austerity measures in Denmark. No gender disparities reported.
*Sub-domain: Income support / social insurance*					
Barr B, Kinderman P*,* et al. 2015. [35] +++^c^	Investigate whether mental health trends increased during a period of recession and welfare reform and whether inequalities existed in these trends.	18–59 years (*N* =2,171,741) Longitudinal (time trends) England	Disability, unemployment & housing benefits	Self-reported poor mental health	Focus: SES (unemployment and low education as a proxy for low SES) Increase in mental health problems were greatest amongst people outside of work and with low education. Increased inequalities following austerity measures and welfare reforms.
Barr B, Taylor-Robinson D*,* et al. 2016. [36] +++^c^	Investigate whether the new UK disability assessment was associated with an increase in poor mental health and whether these changes differed between local authorities.	18–64 years (*N* = 149 local authorities) Longitudinal (natural policy experiment) England	Disability benefits reassessments	Suicide, anti-depressant prescriptions and self-rated mental health *(S)*	Focus: SES inequalities (local area inequalities) Increase in mental health problems associated with change in policy. Greatest increase in mental health problems especially for persons living in most deprived areas. Policy increased health inequalities between different deprived and non-deprived areas.
Blomqvist S, Burström B*,* et al. 2014. [37] +++^c^	Investigate whether health inequalities increased between employed and unemployed women between 2010 compared to 2006 after major Swedish social insurance reforms.	18–64 years (*N*= 24,258) (2006–13,630; 2010–10,268) Repeated cross-sectional Sweden	Social insurance incl. Sickness + unemployment insurance	Mental distress *(GHQ12)*	Focus: SES inequalities (employed vs. unemployed) Mental distress increased in all groups but more so among groups outside the labour market (i.e. lower SES group).
Van der Wel KA, Bambra C*,* et al. 2015. [38] +++	Investigate whether the association between poor working conditions or a low level of education and poor mental health is less in countries providing higher levels of sickness benefit provisions.	25–60 years (working individuals) (*N*=22,504) Cross-sectional 28 European countries^b^	Sickness benefits and working conditions	Mental well-being *(WHO-5)*	Focus: SES inequalities (low education, exposure to psychosocial strain and physically hazardous work) mental well-being was better for those who were exposed to psychosocial job strain and physical hazards or low education in countries with more generous sickness benefit provision. Mental health inequalities were smaller in countries with more generous sickness benefits
Policy domain – Area-based initiatives					
Mohan G, Longo A*,* et al. 2017. [39] +++	Assess the health impacts of a major urban regeneration policy.	Household members aged 16 years and older (*N* = 3458, Wave 1; *N*=1550, Wave 2) Longitudinal (Quasi-experimental design) Northern Ireland	Neighbourhood renewal	Mental distress *(GHQ-12)*	Focus: Gender and SES No discernable impact on mental distress or health inequalities.
Stafford M, Badland H*,* et al. 2014 [40] +++	Determine whether the New Deal for Communities (NDC) intervention contributed towards reducing health inequalities and their social determinants	Men 25–65 yearsWomen 25–60 years (*N* = 109,207) Cross-sectional England	Area-based intervention - New Deal for Communities (NDC)	Mental health and mental distress *(MHI-5 and GHQ-12)*	Focus: SES inequalities No discernable impact on poor mental health between NDC areas and non-NDC areas.
Walthery P, Stafford M*,* et al. 2015 [41] ++	Determine whether the NDC program had an overall effect on mental health over time and whether these changes differ between socio-economic groups.	16 years and older (*N* = 11,648) Longitudinal (Cohort) England	Area-based intervention - NDC	Mental health *(MHI-5)*	Focus: SES inequalities No overall effect of NDC but some evidence that mental health improved for women. Increase in inequality in mental health between low and high socio-economic groups in control group.

+++ High quality study; ++ Medium quality study

*ALMP* Active Labour Market Policies

*ASQ-SE* Ages and Stages Questionnaire

*CDA* Child Development Accounts

*CES-D8* Center for Epidemiological Studies Depression Scale

*GHQ-12* Global Health Questionnaire

*HSBC* Health Behavior in School Age Children

*MCS* Mental Health Component Score

*MHI-5* Mental Health Inventory

*NDC* New Deal for Communities

*NMSC* Neo-Marxian Social Class

*PPL* Paid Parental Leave

*S* Suicide

*SES* Socio-Economic-Status

*SF* Short Form health survey

*SF-36* Short Form health survey (36 items)

*WHO-5* World Health Organization Well-Being Index

^a^Study measured depression only in the third wave of the study, so the sample which included MH consisted of 23 countries and 6603 participants

^b^Includes Sweden

^c^looks at austerity

As results showed that the type of welfare regime strongly influenced the direction of some policy domains, the dimensions of employment policies, family policies and income support and social insurance policies, were added as sub-domains to the welfare state domain. The following section outlines the results by each policy domain or sub-domain.

### Welfare states

Nine articles focused on the policy domain of overall welfare states [21–29], meaning that comparisons were made by categorising European countries into welfare regime types, such as the Nordic dual earner/carer model, family oriented, and market-oriented models. Four of the nine articles addressed gender inequalities, three addressed SES inequalities and two addressed gender and SES. Two articles, (Sekine*,* et al. [26] and Sekine*,* et al. [27]) were based on the same study, but the former focused on SES inequalities while the latter focused on gender inequalities. Only one study focused on social expenditures [29] and the other eight focused on employment or work characteristics within different welfare regimes.

A dual-earner model where both partners contribute to wage earning and care responsibilities (typically in the Nordic countries) seems to be associated with better mental health outcomes for women while the market-oriented model (e.g. the UK) was associated with worse mental health outcomes for women [21–23, 27, 28]. There also appears to be less of a gap between men and women when it comes to mental health functioning in the dual-earner model [23]. Furthermore, greater investment in social spending and family focused welfare models were associated with better mental health outcomes for women [29].

### Family policy

Four articles focused on the policy sub-domain of family [30–33]. One of these focused-on gender inequality while the other three focused on SES inequality. Findings from Huang*,* et al. [32] and Rathmann*,* et al. [33] had opposing conclusions in that Huang*,* et al. [32] found that cash benefits reduced mental health inequalities between children of lone and couple mothers, and Rathmann*,* et al. [33] concluded that an increase in family benefits actually led to a greater gap in SES inequality in mental health outcomes. The limited evidence generally suggests that investment in family benefits leads to overall better mental health outcomes but may not reduce the gap in inequalities in mental health outcomes.

### Employment policy

Only one article focused on the policy sub-domain of employment and addressed gender and SES inequality [34]. As such, we cannot conclude about inequalities in mental health outcomes related to this domain. However, given the quality of the study design and the large sample size, we should consider that in this case, austerity measures contributed to worse mental health outcomes among lower SES groups [34].

### Income support and social insurance

Four studies focused on the policy sub-domain of income support, all of which analysed SES inequalities rather than gender inequalities [35–38]. Three of these articles focused on austerity measures and found that mental health inequalities increased, and particularly vulnerable groups experienced greater mental health problems. Van der Wel*,* et al. [38] found that mental health inequalities were smaller in countries with more generous sickness benefits. It is unclear if these effects are a direct result of the policies or if they work through other mechanisms. For example, Barr*,* et al. [36] suggest that austerity measures may have contributed to increased suicide rates and other mental health problems while Blomqvist*,* et al. [37] conclude that inequalities in mental health among women could be due to stricter eligibility criteria and decrease in benefit levels but there is no definitive evidence that policy change (i.e. tightening eligibility criteria and reduced benefit levels) leads to mental distress. The limited evidence shows that more generous welfare benefits are associated with better mental health outcomes and austerity measures are associated with poorer mental health outcomes including increased suicide rates. Additionally, austerity measures seem to contribute to widening the social inequalities gap.

### Area-based initiatives

Three articles focused on the policy domain of area-based initiatives, or interventions in a specific geographical location [39–41]. Two studies focused on the New Deal for Communities initiative in England [40, 41] with both focusing on SES inequality. Mohan, et al. [39] studied a different area-based initiative and focused on gender and SES inequality. Limited results regarding area-based initiatives show that these interventions can prevent or reduce the gap in social inequalities of mental health, or at least prevent the widening of this gap in the targeted areas, and that neighbourhood renewals in more disadvantaged areas provide some improvement to women’s mental well-being.

### Education

We did not find any articles focused on the policy domain of education which met our criteria. We therefore cannot draw any conclusions related to educational policy and mental health inequalities.

## Discussion

We synthesised the literature examining the impact of structural determinants on mental health inequalities, specifically focusing on economic and social policies underpinning the welfare state, and prevailing societal norms (see Table 4). Of the 21 research articles identified, most were observational studies, and only two studies used a natural policy experiment study design. Of the policy domains examined, welfare states were the most comprehensively researched. We should note that most included studies focusing on welfare states used a regime approach (e.g. Korpi’s [13]) but as Bergqvist*,* et al. [42] argue there are other ways to examine welfare states such as through an institutional or expenditure approach. Other approaches may provide alternative perspectives on our research question.

**Table 4:** Summary of changes in inequality by policy domain

Policy domain	Summary of changes in inequalities
Welfare States	The evidence indicates that the Nordic model was associated with fewer mental health problems and fewer gender inequalities compared to other welfare regimes especially basic-security/market welfare states.
Family	The evidence indicates that welfare regimes with more inclusive family policies may reduce inequalities in mental health outcomes for women.
Employment	The evidence indicates increases in mental health problems for those in lower SES after welfare reforms (austerity measures).
Income Support	The evidence indicates that restrictions on income support may have negative effects (see employment). More generous welfare benefits are associated with fewer SES inequalities in mental health.
Education	Unclear knowledge about this policy domain.
Area-based initiatives	The evidence indicates that neighbourhood renewals in more disadvantaged areas provide some improvement to women’s mental well-being.

This review indicates that more comprehensive and gender inclusive welfare states lead to better mental health outcomes especially for women, but there is little evidence that this reduces socio-economic inequalities. We discuss these issues separately below.

### Gender inequalities and mental health

Evidence from the welfare state domain indicated that dual-earner models (typically found in the Nordic countries) were associated with better mental health outcomes and less prominent mental health inequalities by gender, compared to other welfare regimes especially basic-security/market welfare states [21, 23, 26–28]. These findings align with findings of Borrell*,* et al. [3] that in dual-earner models, public policies support women’s employment while imposing more equitable sharing of domestic work leading to better health outcomes.

Three studies examined the intersection between gender and relationship status [28, 30, 32], highlighting a socially and economically vulnerable group of women; lone mothers. Van de Velde*,* et al. [28] found that, in general, lone mothers’ mental health seems to be worse than cohabitating mothers, aligning with other studies (see for example [43–46]. Included studies looked at welfare state arrangements and tested specific measures to lessen financial strain. Van de Velde*,* et al. [28] conclude that welfare regimes may moderate inequalities in mental health between lone and cohabitating mothers, finding smaller inequalities in Sweden (i.e. Nordic welfare regime) than Britain (i.e. Market-oriented welfare regime). Huang*,* et al. [32] suggests that cash benefits to lone mothers are one way to reduce the gap in mental health between children of lone and cohabitating mothers. However, Bergqvist*,* et al. [42] notes that reducing inequalities takes a combination of generous family benefits and supporting women in the labour market. On the other hand, Whitehead*,* et al. [44] found that the pressure for lone mothers to work in Sweden could contribute to worse health outcomes. Many studies show that family policies facilitate the work-family balance and decrease financial strain, both factors are associated with better health among lone mothers (see for example [44–46], however, many of these studies mostly focus on mothers’ general health, rather than mental health outcomes.

While outside the scope of our article, some included studies [23, 26, 27] emphasised the role that job quality plays in gender differences. For example, De Moortel*,* et al. [23] purposes that differential exposure to bad quality employment (e.g. non-permanent contracts, low wage, lack of union representation) is partly explained by welfare regimes.

### Socio-economic inequalities and mental health

While the Nordic countries seem to produce better mental health outcomes for women, our results do not support that this approach reduces socio-economic inequalities in mental health outcomes. Rather, our results support Mackenbach’s [47] conclusions that strong welfare states such as in Sweden do not buffer against socio-economic inequalities in health.

We found that the evidence on welfare states and socio-economic inequalities was inconsistent. On the one hand, Niedzwiedz*,* et al. [24] suggests that higher spending on active labour market programmes reduced inequalities, specifically by improving mental health outcomes among those with the lowest education. However, Rathmann*,* et al. [33] found that higher spending on social protection, especially during the recent recession, is not enough to reduce the socio-economic inequalities in the mental health of adolescents. Rather, the authors suggest that a combination of social spending *and* programs directly targeting adolescents could be more effective [33]. Hewitt*,* et al. [31] also found that increased spending on paid parental leave, improved overall maternal mental health but did not decrease the gap between mothers with low SES and high SES.

Contrasting these studies, Nordenmark*,* et al. [25] note that socio-economic inequalities could be wider in Sweden than the UK because of a ‘levelling down’ process that happens with those in higher SES in the UK. The authors explain that persons with higher SES experience greater economic strain receiving flat-rate benefits (i.e. the UK) as their drop in income is larger, compared to countries with income replacement (i.e. Sweden), leading to poorer mental health outcomes on flat-rate benefits than persons with lower SES [25].

### Austerity measures associated with poor mental health outcomes

Four of the included studies [34–37] showed an association between poor mental health outcomes and austerity measures – reducing government spending, e.g. by cutting programs and reducing benefit levels. A growing body of literature on the direct and indirect health effects of austerity measures support our findings (see for example [15, 48]). More generous public policies like those in Nordic welfare states are associated with better overall mental health outcomes, even if they do not reduce socio-economic inequalities.

### Why so little on education policy?

We propose that the absence of studies focused on education policy is in part because most school policy and school intervention research focus on *academic* outcomes rather than mental health outcomes. Academic outcomes are more accessible to researchers given that children are already assessed based on their school performances. Academic achievement is closely related to mental health, and the two are associated throughout the life course [49], however, more research is warranted to disentangle some of these mechanisms. Furthermore, school is often seen as a neutral environment that is supposed to ‘level the playing field’ to some extent. The focus of inequality is often based on students’ background characteristics rather than what actually happens at school, or how these factors interact [50]. Other studies examine much older policy changes and do not explicitly focus on mental health outcomes (see for example [51]). Further research on school-level determinants of mental health outcomes and investigation of school systems which have undergone changes in educational policy may help elucidate some of these questions.

### Limitations

Readers should interpret results from this review with caution, given the heterogeneity of the literature with regards to methodology, mental health outcomes and policy settings. Other methodological limitations included inconsistencies in choice of comparison groups, and datasets often lacking sufficient information to comprehensively adjust for confounding factors, both at the individual and area-level.

The generalizability of our study is also somewhat limited by the definitions used. Our mental health definition included all aspects of mental health meaning that our analysis did not capture the nuanced differences, for example, between different mental illnesses or psychiatric comorbidity as opposed to mental well-being [52]. Our definition of structural determinants was also broad encompassing many policies, making policy conclusions challenging. Whitehead*,* et al. [44] suggest that a better approach is to focus on specific groups in the population and particular policies. We deliberately took a broad approach to get an overview, given that no systematic review has examined structural determinants and inequalities of mental health before.

Health inequalities is a growing field of research. Given the potential importance of structural determinants of mental illness, it is surprising that we did not find more research articles assessing this research question. There are clearly methodological challenges in designing studies in this area. We must also note that important variations in study context mean that other factors, such as economic trends, migration trends, and the political climate may have played a role. Furthermore, it is important to acknowledge the time lag from policy implementation to observing any associated effects on mental health [53].

### Implications

To the best of our knowledge, this is the first review to examine structural determinants in inequalities in mental health. Additionally, our study is one of the few that focuses specifically on mental health outcomes rather than inequalities focused on self-rated health outcomes. Our study provides an overview of what limited evidence is available in this field and identifies areas of future research and policy directions.

#### Research

In this review, we identified important gaps in the literature for future research. Area-based initiatives and educational policy for example are understudied. Studies should specifically research inequalities, if we are to increase knowledge in this area. Methodologically, we need more natural policy experiments and more studies utilising historical cohort data to examine effects of structural determinants over a longer time frame. Studies drawing on the life-course approach would also strengthen this area of research, given that the risk of mental illnesses may start as early as in childhood and may accumulate over time. Finally, we must acknowledge that the health care system may be a possible mediator. However, given that most mental health policy research focuses on health care access we intentionally excluded the health care domain from this review to focus on other important policy domains. Future research should integrate the health policy domain.

Research comparing welfare states is important, but we must also compare within welfare states (e.g. [43]) and follow change over time. For example, although the Nordic countries share an overall ethos of equality and a strong focus on gender equality, there are differences between policy designs in each country [46]. As such, more case study research on the different policy designs of Nordic countries are needed before we can conclude that all Nordic countries promote better mental health outcomes for women.

#### Policy

The findings from this review bear some relevance to policy. For instance, our results indicate that austerity measures are associated with poor mental health outcomes and possibly increased suicides [29, 36]. Our findings should be a cautionary tale for governments wanting to shrink welfare states.

Our review also indicates that improving mental health outcomes may present policy-makers with a trade-off between reducing socio-economic inequalities *or* improving overall mental health outcomes. We need more innovative policy solutions that reduce the risk of this trade-off.

## Conclusion

In Europe and elsewhere, rising concern about inequality in health and increased prevalence of mental ill-health, means that ignoring the structural policies that may contribute to inequalities in mental health is no longer an option. This review provides knowledge to policy-makers and researchers when considering reforming policies to reduce inequalities in mental health outcomes. While, this review shows limited evidence supporting the causal effects of structural determinants on socio-economic inequalities in mental health, we found some evidence that policy may affect gender inequalities. The lack of evidence should not be interpreted as lack of effect. To strengthen the evidence base within the structural determinants of mental health inequalities research field and inform policies to reduce inequalities in mental health, future studies should seek to apply innovative methods to overcome the inherent methodological challenges in this area.

## Additional files

Additional file 1: Detailed Search Strategy. (DOCX 37 kb)

Additional file 2: Inclusion/Exclusion Criteria for Review. (DOCX 24 kb)

Additional file 3: Quality Appraisal Tool. (DOCX 23 kb)

Additional file 4: Quality Assessment Scores. (XLSX 19 kb)

## Abbreviations

ALMP: Active Labour Market Policies
ASQ-SE: Ages and Stages Questionnaire
CDA: Child Development Accounts
CES-D8: Centre for Epidemiological Studies Depression Scale
GHQ-12: Global Health Questionnaire
HBSC: Health Behaviour in School-Age Children
HEB-Wales Tool: Health Evidence Bulletin, Wales
MCS: Mental Health Component Score
MH: Mental Health
MHI-5: Mental Health Inventory
MWB: Mental Well-Being
NDC: New Deal for Communities
NMSC: Neo-Marxian Social Class
OECD: Organization for Economic Co-operation and Development
PPL: Paid Parental Leave
PRISMA: Preferred Reporting Items for Systematic Reviews and Meta-Analyses
SES: Socio-Economic Status
SF: Short Form Health Survey
SF-36: Short Form Health Survey (36 items)
SOPHIE: Evaluating the Impact of Structural Policies on Health Inequalities
WHO: World Health Organization

## Footnotes

1 The study by Huang et al. 2017 included mothers as participants but measured mental health on the child level. Thus the exposure was on the adult level and the outcome was on the child level.
