# Supplementary material for: How do macro-level structural determinants affect inequalities in mental health? – a systematic review of the literature
Source: Int J Equity Health. 2018 Dec 6;17:180. doi: 10.1186/s12939-018-0879-9 (PMC6284306; doi:10.1186/s12939-018-0879-9)
Supplement: Supplementary file 1 — Detailed Search Strategy. (DOCX 37 kb) [file 12939_2018_879_MOESM1_ESM.docx]

**Additional File 1. Search strategy**

1. Medline (Ovid)

| Date of Search: 2017-03-16  Number of hits: 1025  Comments: | Field labels: .ti,ab,kf. = title, abstract, keyword exp/ = MeSH, exploded / = MeSH, not exploded adjx = within x words |
| --- | --- |
| 1. Policy/  2. Fiscal Policy/  3. Social control policies/  4. exp Public Policy/  5. Policy making/  6. exp Government/  7. Government Regulation/  8. exp Government Agencies/  9. Government Programs/  10. "legislation and jurisprudence".fs.  11. exp Legislation as Topic/  12. exp Taxes/  13. exp Political Systems/  14. Politics/  15. (capitalism or federal or government* or jurispruden* or law or legislation* or legal or neoliberalis* or policy or structural determinant* or taxes or taxation* or welfare or politic* or power constellation* or retrenchment* or social movement*).ti,ab,kf.  16. or/1-15  17. Social determinants of health/  18. exp Insurance, Disability/  19. exp Public assistance/  20. exp Social Welfare/  21. (disability welfare or dole or income support or old age security).ti,ab,kf.  22. (social adj1 (insurance* or program* or benefit* or security or welfare)).ti,ab,kf.  23. ((disabilit* or food or government* or invalid* or old age or public or welfare) adj3 (allowance* or assistance or benefit* or insurance or payment* or pension or program* or support)).ti,ab,kf.  24. exp Housing/  25. ((home owner* or homebuyer* or home buyer* or mortgage*) adj3 (grant* or subsid* or assistance or voucher*)).ti,ab,kf.  26. (housing adj3 (act or affordab* or assistance or availab* or estates or law or low income or policy or public or quality or regulation* or social or subsid* or tax* or voucher*)).ti,ab,kf.  27. (rent adj3 (assistance or ceiling or control or subsid*)).ti,ab,kf.  28. (homeless* adj3 (prevent* or program* or service* or strateg*)).ti,ab,kf.  29. exp Employment/  30. (active labor market polic* or active labour market polic* or alms or employment* or flexicurity or jobless* or minimum wage* or labor market program* or labour market program* or labor union* or labour union* or workless* or unemploy*).ti,ab,kf.  31. "welfare to work".ti,ab,kf.  32. ((insecur* or precarious) adj3 (employment* or job* or work)).ti,ab,kf.  33. (tax adj2 (credit* or reduc* or subsid*)).ti,ab,kf.  34. Education/  35. exp Education/  36. exp Educational Status/  37. exp Family leave/  38. Child Day Care Centers/  39. Nurseries/  40. (child day care center* or education* or nursery or nurseries or school* or tuition*).ti,ab,kf.  41. ((family or maternal or parental or paternal) adj1 leave).ti,ab,kf.  42. or/17-41  43. Health Status Disparities/  44. Health Equity/  45. (disadvantage* or discrimination* or disparit* or equalit* or equity or equities or inequity or inequities or inequalit* or injustice* or social gradient*).ti,ab,kf.  46. or/43-45  47. Mental disorders/  48. Mental Health/  49. Depression/  50. exp Suicide/  51. exp Anxiety Disorders/  52. exp "Bipolar and Related Disorders"/  53. exp Dissociative Disorders/  54. exp "Feeding and Eating Disorders"/  55. exp Mood Disorders/  56. exp Neurocognitive Disorders/  57. exp Personality Disorders/  58. exp "Schizophrenia Spectrum and Other Psychotic Disorders"/  59. exp Somatoform Disorders/  60. exp Mental Fatigue/  61. exp Stress, Psychological/  62. exp Sleep Wake Disorders/  63. (agoraphobi* or anorectic or anorexia* or anxiety or binge eating or bipolar* or borderline personalit* or bulimi* or depress* or dual personalit* or dyssomnia* or female athlete triad or hypochondri* or insomnia* or mental health or multiple personalit* or neurasthenia* or neuroses or neurotic or neurocirculatory asthenia or panic or paranoi* or parasomnia* or phobi* or pica or psychoses or psychotic or schizoaffect* or schizophren* or schizoid or schizotypal or sleep deprivation or sleep problem* or suicid* or wellbeing or well being).ti,ab,kf.  64. ((affective or appetite or body dysmorphic or body image or cognition or consciousness or conversion or cyclothymic or dissociative or dysthymic or eating or factitious or feeding or hoarding or mental or manic or mood or neurocognitive or neurotic or obsessive compulsive or personality or premenstrual dysphoric or sleep or somatoform) adj1 disorder*).ti,ab,kf.  65. or/47-64  66. exp Europe/  67. exp North America/  68. Australia/  69. New Zealand/  70. (Europe* or Andorra or Australia or Austria or Balkan or Belgium or Canada or France or Germany or Gibraltar or Great Britain or England or Scotland or Wales or Greece or Ireland or Italy or Liechtenstein or Luxembourg or Monaco or Netherlands or New Zealand or Nordic countr* or North america or Portugal or Denmark or Finland or Iceland or Norway or San marino or Scandinavia or Sweden or Spain or Switzerland or United kingdom or United states).ti,ab,kf.  71. or/66-70 | |

2. Embase (Embase.com)

| Date of Search: 2017-03-16  Number of hits: 785  Comments: | Field labels: :ti,ab = title, abstract exp/ = Emtree, exploded NEAR/x = within x words |
| --- | --- |
| #1 'policy'/de  #2 'government regulation'/de  #3 'law'/exp  #4 'tax'/de  #5 'politics'/exp  #6 capitalism:ti,ab OR federal:ti,ab OR government*:ti,ab OR jurispruden*:ti,ab OR law:ti,ab OR legislation*:ti,ab OR legal:ti,ab OR neoliberalis*:ti,ab OR policy:ti,ab OR 'structural determinant*':ti,ab OR taxes:ti,ab OR taxation*:ti,ab OR welfare:ti,ab OR politic*:ti,ab OR 'power constellation*':ti,ab OR retrenchment*:ti,ab OR 'social movement*':ti,ab  #7 #1 OR #2 OR #3 OR #4 OR #5 OR #6  #8 'social determinants of health'/de  #9 'social insurance'/de  #10 'social security'/de  #11 'social care'/de  #12 'social welfare'/de  #13 'disability welfare':ti,ab OR dole:ti,ab OR 'income support':ti,ab OR 'old age security':ti,ab  #14 (social NEAR/1 (insurance* OR program* OR benefit* OR security OR welfare)):ti,ab  #15 (('home owner*' OR homebuyer* OR 'home buyer*' OR mortgage*) NEAR/3 (grant* OR subsid* OR assistance OR voucher*)):ti,ab  #16 housing:ti,ab AND adj3:ti,ab AND (act:ti,ab OR affordab*:ti,ab OR assistance:ti,ab OR availab*:ti,ab OR estates:ti,ab OR law:ti,ab OR low:ti,ab AND income:ti,ab OR policy:ti,ab OR public:ti,ab OR quality:ti,ab OR regulation*:ti,ab OR social:ti,ab OR subsid*or:ti,ab AND tax*:ti,ab OR voucher*:ti,ab)  #17 (rent NEAR/3 (assistance OR ceiling OR control OR subsid*)):ti,ab  #18 (homeless* NEAR/3 (prevent* OR program* OR service* OR strateg*)):ti,ab  #19 'employment'/exp  #20 'workman compensation'/de  #21 'active labor market polic*':ti,ab OR 'active labour market polic*':ti,ab OR alms:ti,ab OR employment*:ti,ab OR flexicurity:ti,ab OR jobless*:ti,ab OR 'minimum wage*':ti,ab OR 'labor market program*':ti,ab OR 'labour market program*':ti,ab OR 'labor union*':ti,ab OR 'labour union*':ti,ab OR workless*:ti,ab OR unemploy*:ti,ab OR 'welfare to work':ti,ab  #22 ((insecur* OR precarious) NEAR/3 (job* OR work)):ti,ab  #23 (tax NEAR/2 (credit* OR reduc* OR subsid*)):ti,ab  #24 'education'/exp  #25 'educational status'/de  #26 'family leave'/exp  #27 'school'/exp  #28 'day care'/de  #29 'child day care center*':ti,ab OR education*:ti,ab OR nursery:ti,ab OR nurseries:ti,ab OR school*:ti,ab OR tuition*:ti,ab  #30 ((family OR maternal OR parental OR paternal) NEAR/1 leave):ti,ab  #31 #8 OR #9 OR #10 OR #11 OR #12 OR #13 OR #14 OR #15 OR #16 OR #17 OR #18 OR #19 OR #20 OR #21 OR #22 OR #23 OR #24 OR #25 OR #26 OR #27 OR #28 OR #29 OR #30  #32 'health disparity'/de  #33 'health equity'/de  #34 disadvantage*:ti,ab OR discrimination*:ti,ab OR disparit*:ti,ab OR equalit*:ti,ab OR equity:ti,ab OR equities:ti,ab OR inequity:ti,ab OR inequities:ti,ab OR inequalit*:ti,ab OR injustice*:ti,ab OR 'social gradient*':ti,ab  #35 #32 OR #33 OR #34  #36 'mental disease'/de  #37 'mental health'/exp  #38 'suicidal behavior'/exp  #39 'anxiety disorder'/exp  #40 'dissociative disorder'/exp  #41 'eating disorder'/exp  #42 'mood disorder'/exp  #43 'disorders of higher cerebral function'/exp  #44'personality disorder'/exp  #45 'psychosis'/exp  #46 'somatoform disorder'/exp  #47 'mental stress'/de  #48 'sleep disorder'/exp  #49 agoraphobi*:ti,ab OR anorectic:ti,ab OR anorexia*:ti,ab OR anxiety:ti,ab OR binge:ti,ab AND eating:ti,ab OR bipolar*:ti,ab OR 'borderline personalit*':ti,ab OR bulimi*:ti,ab OR depress*:ti,ab OR 'dual personalit*':ti,ab OR dyssomnia*:ti,ab OR 'female athlete triad':ti,ab OR hypochondri*:ti,ab OR insomnia*:ti,ab OR 'mental health':ti,ab OR 'multiple personalit*':ti,ab OR neurasthenia*:ti,ab OR neuroses:ti,ab OR neurotic:ti,ab OR 'neurocirculatory asthenia':ti,ab OR panic:ti,ab OR paranoi*:ti,ab OR parasomnia*:ti,ab OR phobi*:ti,ab OR pica:ti,ab OR psychoses:ti,ab OR psychotic:ti,ab OR schizoaffect*:ti,ab OR schizophren*:ti,ab OR schizoid:ti,ab OR schizotypal:ti,ab OR 'sleep deprivation':ti,ab OR 'sleep problem*':ti,ab OR suicid*:ti,ab OR wellbeing:ti,ab OR 'well being':ti,ab  #50 ((affective OR appetite OR 'body dysmorphic' OR 'body image' OR cognition OR consciousness OR conversion OR cyclothymic OR dissociative OR dysthymic OR eating OR factitious OR feeding OR hoarding OR mental OR manic OR mood OR neurocognitive OR neurotic OR 'obsessive compulsive' OR personality OR 'premenstrual dysphoric' OR sleep OR somatoform) NEAR/1 disorder*):ti,ab  #51 #36 OR #37 OR #38 OR #39 OR #40 OR #41 OR #42 OR #43 OR #44 OR #45 OR #46 OR #47 OR #48 OR #49 OR #50  #52 'southern europe'/exp  #53 'western europe'/exp  #54 'north america'/exp  #55 'australia and new zealand'/exp  #56 europe*:ti,ab OR andorra:ti,ab OR australia:ti,ab OR austria:ti,ab OR balkan:ti,ab OR belgium:ti,ab OR canada:ti,ab OR france:ti,ab OR germany:ti,ab OR gibraltar:ti,ab OR 'great britain':ti,ab OR england:ti,ab OR scotland:ti,ab OR wales:ti,ab OR greece:ti,ab OR ireland:ti,ab OR italy:ti,ab OR liechtenstein:ti,ab OR luxembourg:ti,ab OR monaco:ti,ab OR netherlands:ti,ab OR new:ti,ab AND zealand:ti,ab OR 'nordic countr*':ti,ab OR 'north america':ti,ab OR portugal:ti,ab OR denmark:ti,ab OR finland:ti,ab OR iceland:ti,ab OR norway:ti,ab OR 'san marino':ti,ab OR scandinavia:ti,ab OR sweden:ti,ab OR spain:ti,ab OR switzerland:ti,ab OR 'united kingdom':ti,ab OR 'united states':ti,ab  #57 #52 OR #53 OR #54 OR #55 OR #56  #58 #7 AND #31 AND #35 AND #51 AND #57 | |

3. PsycInfo (Ovid)

| Date of Search: 2017-03-16  Number of hits: 435  Comments: | Field labels: .ti,ab,id. = title, abstract, keyword exp/ = MeSH, exploded / = MeSH, not exploded adjx = within x words |
| --- | --- |
| 1. politics/  2. policy making/  3. government policy making/  4. welfare reform/  5. education policy/  6. environmental policy/  7. exp health care policy/  8. government/  9. government agencies/  10. exp laws/  11. exp "law (government)"/  12. taxation/  13. exp political economic systems/  14. (capitalism or federal or government* or jurispruden* or law or legislation* or legal or neoliberalis* or policy or structural determinant* or taxes or taxation* or welfare or politic* or power constellation* or retrenchment* or social movement*).ti,ab,id.  15. or/1-14  16. workers' compensation insurance/  17. medicaid/  18. medicare/  19. social security/  20. "welfare services (government)"/  21. community welfare services/  22. (disability welfare or dole or income support or old age security).ti,ab,id.  23. (social adj1 (insurance* or program* or benefit* or security or welfare)).ti,ab,id.  24. ((disabilit* or food or government* or invalid* or old age or public or unemploy* or welfare) adj3 (allowance* or assistance or benefit* or insurance or payment* or pension or program* or support)).ti,ab,id.  25. exp housing/  26. ((home owner* or homebuyer* or home buyer* or mortgage*) adj3 (grant* or subsid* or assistance or voucher*)).ti,ab,id.  27. (housing adj3 (act or affordab* or assistance or availab* or estates or law or low income or policy or public or quality or regulation* or social or subsid* or tax* or voucher*)).ti,ab,id.  28. (rent adj3 (assistance or ceiling or control or subsid*)).ti,ab,id.  29. (homeless* adj3 (prevent* or program* or service* or strateg*)).ti,ab,id.  30. exp employment status/  31. employment history/  32. supported employment/  33. downsizing/  34. exp personnel termination/  35. job security/  36. (active labor market polic* or active labour market polic* or alms or employment* or flexicurity or jobless* or minimum wage* or labor market program* or labour market program*or labor union* or labour union* or workless* or unemploy*).ti,ab,id.  37. "welfare to work".ti,ab,id.  38. ((insecur* or precarious) adj3 (job* or work)).ti,ab,id.  39. (tax adj2 (credit* or reduc* or subsid*)).ti,ab,id.  40. exp education/  41. exp educational background/  42. literacy/  43. employee leave benefits/  44. child day care/  45. nursery schools/  46. (child day care center* or education* or nursery or nurseries or school* or tuition*).ti,ab,id.  47. ((family or maternal or parental or paternal) adj1 leave).ti,ab,id.  48. or/16-47  49. health disparities/  50. (disadvantage* or discrimination* or disparit* or equalit* or equity or equities or inequity or inequities or inequalit* or injustice* or social gradient*).ti,ab,id.  51. or/49-50  52. mental disorders/  53. mental health/  54. "depression (emotion)"/  55. exp suicide/  56. attempted suicide/  57. suicidal ideation/  58. exp anxiety disorders/  59. exp affective disorders/  60. exp dissociative disorders/  61. exp eating disorders/  62. feeding disorders/  63. exp amnesia/  64. cognitive impairment/  65. huntingtons disease/  66. consciousness disturbances/  67. delirium/  68. exp dementia/  69. dyslexia/  70. personality disorders/ or exp mental disorders/  71. exp psychosis/  72. exp somatoform disorders/  73. compassion fatigue/  74. stress/ or chronic stress/ or environmental stress/ or financial strain/ or exp occupational stress/ or psychological stress/ or social stress/ or exp stress reactions/  75. exp sleep disorders/  76. sleep deprivation/  77. sleep apnea/  78. (agoraphobi* or anorectic or anorexia* or anxiety or binge eating or bipolar* or borderline personalit* or bulimi* or depress* or dual personalit* or dyssomnia* or female athlete triad or hypochondri* or insomnia* or mental health or multiple personalit* or neurasthenia* or neuroses or neurotic or neurocirculatory asthenia or panic or paranoi* or parasomnia* or phobi* or pica or psychoses or psychotic or schizoaffect* or schizophren* or schizoid or schizotypal or sleep deprivation or sleep problem* or suicid* or wellbeing or well being).ti,ab,id.  79. ((affective or appetite or body dysmorphic or body image or cognition or consciousness or conversion or cyclothymic or dissociative or dysthymic or eating or factitious or feeding or hoarding or mental or manic or mood or neurocognitive or neurotic or obsessive compulsive or personality or premenstrual dysphoric or sleep or somatoform) adj1 disorder*).ti,ab,id.  80. or/52-79  81. (Europe* or Andorra or Australia or Austria or Balkan or Belgium or Canada or France or Germany or Gibraltar or Great Britain or England or Scotland or Wales or Greece or Ireland or Italy or Liechtenstein or Luxembourg or Monaco or Netherlands or New Zealand or Nordic countr* or North america or Portugal or Denmark or Finland or Iceland or Norway or San marino or Scandinavia or Sweden or Spain or Switzerland or United kingdom or United states).ti,ab,id.  82. 15 and 48 and 51 and 80 and 81 | |

4. Web of Science

| Date of Search: 2017-03-16  Number of hits: 896  Comments: | Field labels: TOPIC = title, abstract, keywords NEAR/x = within x words |
| --- | --- |
| #1 TOPIC: (capitalism OR federal OR government* OR jurispruden* OR law OR legislation* OR legal OR neoliberalis* OR policy OR "structural determinant*" OR taxes OR taxation* OR welfare OR politic* OR "power constellation*" OR retrenchment* OR "social movement*")  #2 TOPIC: ("disability welfare" OR dole OR "income support" OR "old age security")) *OR* TOPIC: ((social NEAR/1 (insurance* OR program* OR benefit* OR security OR welfare))) *OR* TOPIC: (((disabilit* OR food OR government* OR invalid* OR "old age" OR public OR welfare) NEAR/3 (allowance* OR assistance OR benefit* OR insurance OR payment* OR pension OR program* OR support))) *OR* TOPIC: ((("home owner*" OR homebuyer* OR "home buyer*" OR mortgage*) NEAR/3 (grant* OR subsid* OR assistance OR voucher*))) *OR* TOPIC: ((housing NEAR/3 (act OR affordab* OR assistance OR availab* OR estates OR law OR "low income" OR policy OR public OR quality OR regulation* OR social OR subsid* OR tax* OR voucher*))) *OR* TOPIC: ((rent NEAR/3 (assistance OR ceiling OR control OR subsid*))) *OR* TOPIC: ((homeless* NEAR/3 (prevent* OR program* OR service* OR strateg*))) *OR* TOPIC: (("active labOR market polic*" OR "active labour market polic*" OR alms OR employment* OR flexicurity OR jobless* OR "minimum wage*" OR "labor market program*" OR "labour market program*" OR "labor union*" OR "labour union*" OR workless* OR unemploy*)) *OR* TOPIC: (((insecur* OR precarious) NEAR/3 (employment* OR job* OR work))) *OR* TOPIC: ((tax NEAR/2 (credit* OR reduc* OR subsid*))) *OR* TOPIC: (("child day care center*" OR education* OR nursery OR nurseries OR school* OR tuition*)) *OR* TOPIC: (((family OR maternal OR parental OR paternal) NEAR/1 leave)  #3 TOPIC: (disadvantage* OR discrimination* OR disparit* OR equalit* OR equity OR equities OR inequity OR inequities OR inequalit* OR injustice* OR "social gradient*")  #4 TOPIC: (agoraphobi* OR anorectic OR anorexia* OR anxiety OR "binge eating" OR bipolar* OR "borderline personalit*" OR bulimi* OR depress* OR "dual personalit*" OR dyssomnia* OR "female athlete triad" OR hypochondri* OR insomnia* OR "mental health" OR "multiple personalit*" OR neurasthenia* OR neuroses OR neurotic OR "neurocirculatory asthenia" OR panic OR paranoi* OR parasomnia* OR phobi* OR pica OR psychoses OR psychotic OR schizoaffect* OR schizophren* OR schizoid OR schizotypal OR "sleep deprivation" OR "sleep problem*" OR suicid* OR wellbeing OR "well being")) *OR* TOPIC: (((affective OR appetite OR "body dysmorphic" OR "body image" OR cognition OR consciousness OR conversion OR cyclothymic OR dissociative OR dysthymic OR eating OR factitious OR feeding OR hoarding OR mental OR manic OR mood OR neurocognitive OR neurotic OR "obsessive compulsive" OR personality OR "premenstrual dysphoric" OR sleep OR somatoform) NEAR/1 disorder*)  #5 TOPIC: (Europe* OR Andorra OR Australia OR Austria OR Balkan OR Belgium OR Canada OR France OR Germany OR Gibraltar OR Great Britain OR England OR Scotland OR Wales OR Greece OR Ireland OR Italy OR Liechtenstein OR Luxembourg OR Monaco OR Netherlands OR New Zealand OR Nordic countr* OR North america OR Portugal OR Denmark OR Finland OR Iceland OR Norway OR San marino OR Scandinavia OR Sweden OR Spain OR Switzerland OR United kingdom OR United states)  #6 #5 AND #4 AND #3 AND #2 AND #1 | |

5. Sociological Abstracts (ProQuest)

| Date of Search: 2017-03-17  Number of hits: 270  Comments: | Field labels: TI,AB = title SU.EXACT = Subject heading NEAR/x = within x words |
| --- | --- |
| ((SU.EXACT("Agricultural Policy" OR "Criminal Justice Policy" OR "Cultural Arts Policy" OR "Development Policy" OR "Economic Policy" OR "Educational Policy" OR "Energy Policy" OR "Environmental Policy" OR "Family Policy" OR "Fiscal Policy" OR "Foreign Policy" OR "Government Policy" OR "Health Care Services Policy" OR "Health Policy" OR "Housing Policy" OR "Immigration Policy" OR "Labor Policy" OR "Language Policy" OR "Personnel Policy" OR "Policy" OR "Population Policy" OR "Protectionism" OR "Public Policy" OR "Science Policy" OR "Social Policy" OR "Technology Policy" OR "Telecommunications Policy" OR "Urban Policy" OR "Welfare Policy" OR "Social control" OR "Government Regulation" OR "Central Government" OR "Federal Government" OR "Local Government") OR "Governmentality" OR "Foreign Policy" OR "Government Policy" OR "Immigration Policy" OR "Language Policy" OR "Defense Spending" OR "Government Spending" OR "Central Government" OR "Federal Government" OR "Government" OR "Local Government" OR "Federal Government" OR "Government Agencies" OR "Legislation" OR "Statutes" OR "Taxation" OR "Chieftaincies" OR "Democracy" OR "Dictatorship" OR "Empires" OR "Gerontocracy" OR "Monarchy" OR "Oligarchy" OR "Political Systems" OR "Polyarchy" OR "Republics" OR "Technocracy") OR(TI,AB("capitalism" OR "federal" OR "government*" OR "jurispruden*" OR "law" OR "legislation*" OR "legal" OR "neoliberalis*" OR "policy" OR "structural determinant*" OR "taxes" OR "taxation*" OR "welfare" OR "politic*" OR "power constellation*" OR "retrenchment*" OR "social movement*")))  AND  (((SU.EXACT("Health Insurance" OR "Workers Compensation Insurance" OR "Social Security" OR "Social Welfare")) OR (TI,AB("disability welfare" OR "dole" OR "income support" OR "old age security")) OR (TI,AB(("social*") NEAR/3 ("insurance*" OR "program*" OR "benefit*" OR "security" OR "welfare"))) OR (TI,AB((("disabilit*" OR "food" OR "government*" OR "invalid*" OR "old age" OR "public" OR "unemploy*" OR "welfare") NEAR/3 ("allowance*" OR "assistance" OR "benefit*" OR "insurance" OR "payment*" OR "pension*" OR "program*" OR "support")))))  OR  ((SU.EXACT("Housing Costs" OR "Rents" OR "Housing" OR "Public Housing" OR "Rental Housing" OR "Housing Market" OR "Housing Policy" OR "Public Housing" OR "Rental Housing")) OR (TI,AB(("home owner*" OR "homebuyer*" OR "home buyer*" OR "mortgage*") NEAR/3 ("grant*" OR "subsid*" OR "assistance" OR "voucher*"))) OR (TI,AB(("housing") NEAR/3 ("act" OR "affordab*" OR "assistance" OR "availab*" OR "estates" OR "law" OR "low income" OR "policy" OR "public" OR "quality" OR "regulation*" OR "social" OR "subsid*" OR "tax*" OR "voucher*"))) OR (TI,AB(("rent") NEAR/3 ("assistance" OR "ceiling" OR "control" OR "subsid*"))) OR (TI,AB(("homeless*") NEAR/3 ("prevent*" OR "program*" OR "service*" OR "strateg*"))))  OR  ((SU.EXACT("Employment" OR "Job Security" OR "Multiple Jobholding" OR "Part Time Employment" OR "Self Employment" OR "Temporary Employment" OR "Underemployment" OR "Unemployment" OR "Youth Employment")) OR (TI,AB("active labor market polic*" OR "active labour market polic*" OR "alms" OR "employment*" OR "employment protection" OR "flexicurity" OR "jobless*" OR "minimum wage*" OR "labor market program*" OR "labour market program*" OR "labor union*" OR "labour union*" OR "workless*" OR "unemploy*" OR "welfare to work*")) OR (TI,AB(("insecur*" OR "precarious") NEAR/3 ("employment*" OR "job*" OR "work"))) OR (TI,AB(("tax") NEAR/2 ("credit*" OR "reduc*" OR "subsid*"))))  OR  ((SU.EXACT("Adult Education" OR "Bilingual Education" OR "Coeducation" OR "Distance Education" OR "Doctoral Programs" OR "Education" OR "Elementary Education" OR "Health Education" OR "Higher Education" OR "International Studies" OR "Marriage and Family Education" OR "Masters Programs" OR "Moral Education" OR "Multicultural Education" OR "Physical Education" OR "Postdoctoral Programs" OR "Preschool Education" OR "Primary Education" OR "Religious Education" OR "Rural Education" OR "Secondary Education" OR "Sex Education" OR "Social Science Education" OR "Social Studies" OR "Social Work Education" OR "Sociology Education" OR "Special Education" OR "Teacher Education" OR "Undergraduate Programs" OR "Urban Education" OR "Vocational Education" OR "Womens Education" OR "Educational Inequality" OR "Educational systems" OR "Family Work Relationship" OR "Child Care Services")) OR ((TI,AB("child day care center*" OR "child day care centre*" OR "education*" OR "nursery" OR "nurseries" OR "school*" OR "tuition*")) OR (TI,AB(("family" OR "maternal" OR "parental" OR "paternal") NEAR/1 ("leave"))))))  AND  ((SU.EXACT("Educational Inequality" OR "Income Inequality" OR "Inequality" OR "Sexual Inequality" OR "Social Inequality")) OR (TI,AB("disadvantage*" OR "discrimination*" OR "disparit*" OR "equalit*" OR "equity" OR "equities" OR "inequity" OR "inequities" OR "inequalit*" OR "injustice*" OR "social gradient*")))  AND  ((SU.EXACT("Community Mental Health" OR "Mental Health" OR "Mental Illness" OR "Paranoia" OR "Psychosis" OR "Schizophrenia" OR "Suicide" OR "Anxiety" OR "Affective Illness" OR "Depression (Psychology)" OR "Anorexia Nervosa" OR "Bulimia" OR "Eating Disorders" OR "Forensic Psychiatry" OR "Psychiatry" OR "Social Psychiatry" OR "Fatigue" OR "Psychological Stress" OR "Sleep")) OR (TI,AB("agoraphobi*" OR "anorectic" OR "anorexia*" OR "anxiety" OR "binge eating" OR "bipolar*" OR "borderline personalit*" OR "bulimi*" OR "depress*" OR "dual personalit*" OR "dyssomnia*" OR "female athlete triad" OR "hypochondri*" OR "insomnia*" OR "mental health" OR "multiple personalit*" OR "neurasthenia*" OR "neuroses" OR "neurotic" OR "neurocirculatory asthenia" OR "panic" OR "paranoi*" OR "parasomnia*" OR "phobi*" OR "pica" OR "psychoses" OR "psychotic" OR "schizoaffect*" OR "schizophren*" OR "schizoid" OR "schizotypal" OR "sleep deprivation" OR "sleep problem*" OR "suicid*" OR "wellbeing" OR "well being")) OR (TI,AB(("affective" OR "appetite" OR "body dysmorphic" OR "body image" OR "cognition" OR "consciousness" OR "conversion" OR "cyclothymic" OR "dissociative" OR "dysthymic" OR "eating" OR "factitious" OR "feeding" OR "hoarding" OR "mental" OR "manic" OR "mood" OR "neurocognitive" OR "neurotic" OR "obsessive compulsive" OR "personality" OR "premenstrual dysphoric" OR "sleep" OR "somatoform") NEAR/1 "disorder*")))  AND  ((SU.EXACT("European Union")) OR (TI,AB("Europe*" OR "Andorra" OR "Australia*" OR "Austria*" OR "Balkan" OR "Belgium" OR "Canada" OR "France" OR "German*" OR "Gibraltar" OR "Great Britain" OR "England" OR "Scotland" OR "Wales" OR "Greece" OR "Ireland" OR "Italy" OR "Liechtenstein" OR "Luxembourg" OR "Monaco" OR "Netherlands" OR "New Zealand" OR "Nordic countr*" OR "North america*" OR "Portugal" OR "Denmark" OR "Finland" OR "Iceland" OR "Norway" OR "San marino" OR "Scandinavia*" OR "Sweden" OR "Spain" OR "Switzerland" OR "Swiss" OR "United kingdom" OR "British" OR "United states" OR "USA"))) | |

6. Eric (ProQuest)

| Date of Search: 2017-03-16  Number of hits: 306  Comments: | Field labels: |
| --- | --- |
| ((SU.EXACT.EXPLODE("Policy" OR "Government (Administrative Body)" OR "Legislation" OR "Taxes" OR "Politics") OR (TI,AB("capitalism" OR "federal" OR "government*" OR "jurispruden*" OR "law" OR "legislation*" OR "legal" OR "neoliberalis*" OR "policy" OR "structural determinant*" OR "taxes" OR "taxation*" OR "welfare" OR "politic*" OR "power constellation*" OR "retrenchment*" OR "social movement*")))  AND  (((SU.EXACT.EXPLODE("Health Insurance" OR "Financial support" OR "Social Welfare")) OR (TI,AB("disability welfare" OR "dole" OR "income support" OR "old age security")) OR (TI,AB(("social*") NEAR/3 ("insurance*" OR "program*" OR "benefit*" OR "security" OR "welfare"))) OR (TI,AB((("disabilit*" OR "food" OR "government*" OR "invalid*" OR "old age" OR "public" OR "unemploy*" OR "welfare") NEAR/3 ("allowance*" OR "assistance" OR "benefit*" OR "insurance" OR "payment*" OR "pension*" OR "program*" OR "support")))))  OR  ((SU.EXACT.EXPLODE("Housing" OR "Housing needs" OR "Public housing" OR "Housing Discrimination")) OR (TI,AB(("home owner*" OR "homebuyer*" OR "home buyer*" OR "mortgage*") NEAR/3 ("grant*" OR "subsid*" OR "assistance" OR "voucher*"))) OR (TI,AB(("housing") NEAR/3 ("act" OR "affordab*" OR "assistance" OR "availab*" OR "estates" OR "law" OR "low income" OR "policy" OR "public" OR "quality" OR "regulation*" OR "social" OR "subsid*" OR "tax*" OR "voucher*"))) OR (TI,AB(("rent") NEAR/3 ("assistance" OR "ceiling" OR "control" OR "subsid*"))) OR (TI,AB(("homeless*") NEAR/3 ("prevent*" OR "program*" OR "service*" OR "strateg*"))))  OR  ((SU.EXACT.EXPLODE("Employment" OR "Job Security" OR "Multiple Employment" OR "Part Time Employment" OR "Self Employment" OR "Temporary Employment" OR "Underemployment" OR "Unemployment" OR "Youth Employment")) OR (TI,AB("active labor market polic*" OR "active labour market polic*" OR "alms" OR "employment*" OR "employment protection" OR "flexicurity" OR "jobless*" OR "minimum wage*" OR "labor market program*" OR "labour market program*" OR "labor union*" OR "labour union*" OR "workless*" OR "unemploy*" OR "welfare to work*")) OR (TI,AB(("insecur*" OR "precarious") NEAR/3 ("employment*" OR "job*" OR "work"))) OR (TI,AB(("tax") NEAR/2 ("credit*" OR "reduc*" OR "subsid*"))))  OR  ((SU.EXACT.EXPLODE("Education")) OR ((TI,AB("child day care center*" OR "child day care centre*" OR "education*" OR "nursery" OR "nurseries" OR "school*" OR "tuition*")) OR (TI,AB(("family" OR "maternal" OR "parental" OR "paternal") NEAR/1 ("leave"))))))  AND  ((SU.EXACT.EXPLODE("Inequalities" OR "Equal education")) OR (TI,AB("disadvantage*" OR "discrimination*" OR "disparit*" OR "equalit*" OR "equity" OR "equities" OR "inequity" OR "inequities" OR "inequalit*" OR "injustice*" OR "social gradient*")))  AND  ((SU.EXACT.EXPLODE("Mental Health" OR "Mental Disorders" OR "Psychiatry" OR "Psychology" OR "Psychological patterns" OR "Suicide" OR "Self destructive behavior")) OR (TI,AB("agoraphobi*" OR "anorectic" OR "anorexia*" OR "anxiety" OR "binge eating" OR "bipolar*" OR "borderline personalit*" OR "bulimi*" OR "depress*" OR "dual personalit*" OR "dyssomnia*" OR "female athlete triad" OR "hypochondri*" OR "insomnia*" OR "mental health" OR "multiple personalit*" OR "neurasthenia*" OR "neuroses" OR "neurotic" OR "neurocirculatory asthenia" OR "panic" OR "paranoi*" OR "parasomnia*" OR "phobi*" OR "pica" OR "psychoses" OR "psychotic" OR "schizoaffect*" OR "schizophren*" OR "schizoid" OR "schizotypal" OR "sleep deprivation" OR "sleep problem*" OR "suicid*" OR "wellbeing" OR "well being")) OR (TI,AB(("affective" OR "appetite" OR "body dysmorphic" OR "body image" OR "cognition" OR "consciousness" OR "conversion" OR "cyclothymic" OR "dissociative" OR "dysthymic" OR "eating" OR "factitious" OR "feeding" OR "hoarding" OR "mental" OR "manic" OR "mood" OR "neurocognitive" OR "neurotic" OR "obsessive compulsive" OR "personality" OR "premenstrual dysphoric" OR "sleep" OR "somatoform") NEAR/1 "disorder*")))  AND  ((SU.EXACT.EXPLODE("European Union")) OR (TI,AB("Europe*" OR "Andorra" OR "Australia*" OR "Austria*" OR "Balkan" OR "Belgium" OR "Canada" OR "France" OR "German*" OR "Gibraltar" OR "Great Britain" OR "England" OR "Scotland" OR "Wales" OR "Greece" OR "Ireland" OR "Italy" OR "Liechtenstein" OR "Luxembourg" OR "Monaco" OR "Netherlands" OR "New Zealand" OR "Nordic countr*" OR "North america*" OR "Portugal" OR "Denmark" OR "Finland" OR "Iceland" OR "Norway" OR "San marino" OR "Scandinavia*" OR "Sweden" OR "Spain" OR "Switzerland" OR "Swiss" OR "United kingdom" OR "British" OR "United states" OR "USA")))) | |
