# Supplementary material for: How do macro-level structural determinants affect inequalities in mental health? – a systematic review of the literature
Source: Int J Equity Health. 2018 Dec 6;17:180. doi: 10.1186/s12939-018-0879-9 (PMC6284306; doi:10.1186/s12939-018-0879-9)
Supplement: Supplementary file 2 — Inclusion/Exclusion Criteria for Review. (DOCX 24 kb) [file 12939_2018_879_MOESM2_ESM.docx]

**Additional File 2. Inclusion/Exclusion criteria for review**

|  | Inclusion | Exclusion |
| --- | --- | --- |
| Language | English or Swedish | All other languages |
| Publication  Type | - Original studies from the peer-reviewed literature - Published or in-press | - Studies that have not been peer-reviewed (e.g., grey literature reports, dissertations) - Any work that is not an original research study, including letters, editorials, commentaries, conference proceedings, books and book chapters, meeting abstracts, lectures and addresses - Narrative reviews, systematic reviews, meta-analyses are not eligible, but relevant reviews will be flagged |
| Year of  Publication | - 1996-2015 | - Prior to 1995 |
| Context | - Nordic countries, Western Europe, North America, Australia and New Zealand - *If a study compares countries outside of the chosen context, at least two countries from the above should be included in the study. | - All other countries |
| Study Design | - Quantitative study designs (e.g., natural experiments, country/region comparisons, longitudinal, and multilevel - Use of validated instruments to identify mental disorders/health (e.g., GHQ12, WHO-5 Well-being index) - Results must be compared across time or contexts | - Qualitative study designs |
| Population | - 0 – 64 years (stratified by inequalities e.g., gender, SES or ethnicity | - Older persons (65 years and older) |
| Intervention/  Exposure | - One of the five policy domains: - Employment - Education - Housing - Income support/Social insurance - Family policy   *Intervention must have been introduced after 1990 | - Health care and immigration but relevant studies will be flagged |
| Outcome | - Clinically diagnosed mental disorders. In this study, mental disorders include addiction, common mental disorders (CMD) such as anxiety-based disorders and depression as well as more severe mental disorders (SMD) such as bi-polar disorder and schizophrenia. - Positive (self-rated) mental health - Poor (self-rated) mental health - Rates of suicide | - Gaming addiction - Intellectual disabilities - Dementia |
