# Supplementary material for: How do macro-level structural determinants affect inequalities in mental health? – a systematic review of the literature
Source: Int J Equity Health. 2018 Dec 6;17:180. doi: 10.1186/s12939-018-0879-9 (PMC6284306; doi:10.1186/s12939-018-0879-9)
Supplement: Supplementary file 3 — Quality Appraisal Tool. (DOCX 23 kb) [file 12939_2018_879_MOESM3_ESM.docx]

**Additional File 3. Quality Appraisal Tool**

**INSTRUCTIONS:** Please complete the following template for all articles that were deemed eligible in Level 2 – Full-text screening. Please use the following to score each article: Yes = 2; Can’t tell = 1 and No = 0.

**Reviewer’s name:**

**Paper details:**

| **Authors:** |  |
| --- | --- |
| **Title:** |  |
| **Journal:** |  |

**Part A: What is this paper about?**

|  | **YES** | **CAN’T TELL** | **NO** |
| --- | --- | --- | --- |
| 1. Does the paper address a clearly focused issue?   In terms of:   - the population studied? - (case-control study only) Is the case definition explicit and confirmed? - the outcomes considered? - are the aims of the investigation clearly stated? |  |  |  |

**Part B: Do I trust it?**

|  | **YES** | **CAN’T TELL** | **NO** |
| --- | --- | --- | --- |
| 1. Is the choice of study method appropriate? |  |  |  |
| 1. Is the population studied appropriate?  - (cohort study) Was an appropriate control group used – i.e. were groups comparable on important confounding factors? - (case-control study) Were the controls randomly selected from the same population as the cases? |  |  |  |
| 1. Is confounding and bias considered?  - Have all possible explanations of the effects been considered? - (cohort study) Were the assessors blind to the different groups? - (cohort study) Could selective drop out explain the effect? - (case-control study) How comparable are the same cases and controls with respect to potential confounding factors? - (case-control study) Were interventions and other exposures assessed in the same way for cases and controls? - (case-control study) Is it possible that overmatching has occurred in that cases and controls were matched on factors related to exposure? |  |  |  |
| 1. (Cohort study) Was follow up for long enough?  - Could all likely effects have appeared in the time scale? - Could the effect be transitory? - Was follow up sufficiently complete? - Was dose response demonstrated? |  |  |  |

**Part C: What did they find?**

|  | **YES** | **CAN’T TELL** | **NO** |
| --- | --- | --- | --- |
| 1. Are tables/graphs adequately labelled and understandable? |  |  |  |
| 1. Are you confident with the authors’ choice and use of statistical methods, if employed? |  |  |  |
| 1. What are the results of this piece of research?’  - Are the authors’ conclusions adequately supported by the information cited? |  |  |  |

**Part D: Are results relevant locally?**

|  | **YES** | **CAN’T TELL** | **NO** |
| --- | --- | --- | --- |
| 1. Can the results be applied to the local situation?   Consider difference between the local and study populations (e.g. cultural, geographical, ethical) which could affect the relevance of the study. i.e. is it a pragmatic design or are results only relevant under ‘ideal’ conditions? |  |  |  |
| 1. Were all important outcomes/results considered? |  |  |  |

**Part E: Does the study meet our specific criteria?**

|  | **YES** | **CAN’T TELL** | **NO** |
| --- | --- | --- | --- |
| 1. Does the study use a validated measurement of mental health |  |  |  |
| 1. Can you identify the policy domain that the study examines? |  |  |  |
| 1. Does the study explicitly describe a relevant policy or policies?  - Are details about the policy provided in the introduction or methods section of the study?   **Note: If the policy is not mentioned until the discussion section, please select “no” for this question.** |  |  |  |
| 1. Does the study describe a policy change OR compare a policy across different countries? |  |  |  |
| 1. Does the author(s) explore inequalities related to SES or gender in the results and/or discussion section of the study?  - The study should indicate if inequalities increased, decreased or remained neutral |  |  |  |

**Final Score:**  /30

| **Comments:** |
| --- |
